# Genetic analysis of *RNF2*13 p.R4810K variant in non-moyamoya intracranial artery stenosis/occlusion disease in a Chinese population

**DOI:** 10.1186/s12199-017-0649-0

**Published:** 2017-04-24

**Authors:** Tong Zhang, Congrong Guo, Xin Liao, Jian Xia, XiaoXiao Wang, Jing Deng, Junxia Yan

**Affiliations:** 1 Department of Neurology, the Second Hospital of Hebei Medical University, Heping West Road, Xinhua District, Shijiazhuang, 050000 China; 2 Department of Ophthalmology, the Second Hospital of Hebei Medical University, Heping West Road, Xinhua District, Shijiazhuang, 050000 China; 3 Department of Epidemiology and Health Statistics, XiangYa School of Public Health, Central South University, Shang Mayuanling, KaiFu District, Changsha, 410078 China; 4 Department of Neurology, XiangYa Hospital, Central South University, 87 Xiangya Road, Changsha, Hunan 410008 China

**Keywords:** Intracranial artery stenosis/occlusion disease, Genetics, *RNF213*, p.R4810K, China

## Abstract

**Objectives:**

*RNF213* p.R4810K was identified as a susceptibility variant for moyamoya disease in Asia and non-moyamoya intracranial artery stenosis/occlusion disease in Japan and Korea recently. The occurrence of this variant was evaluated in patients with non-moyamoya intracranial artery stenosis/occlusion disease in China.

**Methods:**

Two study populations were used in this study. One was recruited from the Second Hospital of Hebei Medical University from April 2015 to May 2016. The other was the archived DNA samples of intracranial artery stenosis/occlusion patients in XiangYa Hospital collected in 2014. The occurrence of *RNF213* p.R4810K was investigated in a total of 715 patients with non-moyamoya intracranial artery stenosis/occlusion disease. The carrier rate of *RNF213* p.R4810K in 507 normal individuals was used as control.

**Results:**

Six of 715 patients (0.84%) with non-moyamoya intracranial artery stenosis/occlusion disease and 2 of the 507 normal controls (0.39%) had *RNF213* p.R4810K variant. The carrier rate of *RNF213* p.R4810K was higher in non-moyamoya intracranial artery stenosis/occlusion group than that in the normal group. However, no statistically significant association was observed (Odds ratio, 2.14; 95% confidence interval, 0.43–10.63; *p* = 0.56).

**Conclusions:**

The carrier rate of *RNF213* p.R4810K in Chinese non-moyamoya intracranial artery stenosis/occlusion disease patients was significantly lower than that in Korea or Japan. Genetic heterogeneity was highly indicated. Further systematic genetic epidemiology studies with emphasis on Chinese-specific genetic variants and environmental risk factors of intracranial artery stenosis/occlusion disease in larger population are needed.

## Introduction

The ring finger protein 213 (*RNF213*) was identified as a susceptibility gene for moyamoya disease (MMD) recently [1, 2]. Several studies revealed high frequencies of the same *RNF213* variant- p.R4810K (rs112735431, GenBank accession number AB537889) in East Asian MMD patients compared to normal controls (ORs > 100), which was illustrated as a founder mutation in Japanese, Korean and Chinese patients [1–9]. Indeed, in Japan and Korea, the majority (~80%) of MMD patients carried at least one allele of *RNF213* p.R4810K, which was significantly higher than that in the general population [1, 2, 4–6, 8–16]. In China, the carrier rate of *RNF213* p.R4810K in MMD patients was about 20% [1, 3, 17–19]. MMD risk was highly increased by this variant in the Asian population.

*RNF213* located in chromosome 17q25.3, encoding a 596 kDa protein which functions both as an AAA‑type ATPase and an E3 ligase [20]. AAA-ATPases mediate various cell functions, including membrane fusion/transport, proteolysis, protein disaggregation/refolding, DNA recombination/repair and mitosis/meiosis [21]. AAA-ATPase dysfunction can cause several diseases, such as *PEX1/PEX6* mutations cause multiple organ degeneration [22, 23] and *Cdc48* mutations cause amyotrophic lateral sclerosis [24, 25]. E3 ligase activity may play a role in protein degradation or signaling processes [1]. Even the complete physiological functions of *RNF213* are still unknown, knockdown of *RNF213* in zebrafish leads to abnormal sprouting and irregular diameter of intracranial vessels, suggesting some contribution to vascular formation [1]. Previous studies revealed that a wide spectrum of phenotypes could occur within a family unit despite the members having the identical p.R4810K variant, with some individuals showing the typical phenotype of MMD such as bilateral stenosis/occlusion of the terminal portion of the internal carotid arteries, some showing only unilateral or middle cerebral artery stenosis/occlusion, and others with no abnormalities [1]. Miyawaki et al. reported that a particular subset of Japanese intracranial major artery stenosis/occlusion (ICASO) mainly diagnosed as atherosclerosis not MMD, associated with *RNF213* p.R4810K variant (odds ratio, 16.8; 95% confidence interval, 3.81–74.5; *p* < 0.0001), suggesting that *RNF213* p.R4810K variant might cause various severities of ICASO [4, 6]. Recently, Bang et al. also reported that *RNF213* p.R4810K is a susceptibility variant not only for MMD but also for ICASO in Korean (odds ratio, 22.3; 95% confidence interval, 3.0–164.1; *p* < 0.0001) [13]. ICASO is an important and the most frequent cause of cerebral ischemic stroke among patients of Asian ancestry [26]. Liu et al. explored the association of *RNF213* p.R4810K with MMD in Chinese population [3, 5, 17–19], however, no association data was available about this variant with ICASO in China.

The aim of present study was to verify the generalizability of previous findings in Japan and Korea, investigating the association of *RNF213* p.R4810K variant with ICASO not diagnosed as MMD in a Chinese population, compared with the occurrence of normal individuals as control group.

## Materials and methods

### Study population

The study population was mainly recruited from the Department of Neurology of Hebei Medical University from October 2015 to May 2016. All the patients with ICASO in the absence of MMD who agreed to participate in this study in this period were enrolled (totally 615). In addition, another 100 ICASO patients in XiangYa Hospital of Central South University who had complete clinical information and archived DNA samples also included in this study. The carrier rate of *RNF213* p.R4810K in 507 normal individuals published in other study was used as control [3]. This study was approved by the Medical Ethics Committee of Central South University and the survey participants gave informed consent before the interview and blood samples were taken.

### Diagnosis of ICASO

Participants were diagnosed as non-moyamoya ICASO when they experienced focal or lateralizing symptoms and showed ≥50% stenosis or occlusion at terminal and/or proximal portions of the intracranial major arteries without abnormal vascular networks in the basal ganglia on conventional angiography or MRA which was required by MMD diagnosed criteria [27]. The angiography images were interpreted by ≥2 physicians, including at least 1 radiologist and 1 neurological physician. Clinical information, including age, gender, vascular risk factors such as smoking, alcohol consuming, disease histories of hypertension, diabetes, hyperlipemia was collected (these diseases were defined as self-reported physician diagnosis or pharmaceutical treatment). Patients with potential sources of cardioaortic embolism, other stroke mechanisms such as coagulopathy, vasculitis, arterial dissection or incomplete evaluations were excluded.

### Identification of *RNF213* p.R4810K variant

Peripheral blood samples were obtained from all enrolled patients. Genomic DNA was extracted from the peripheral blood leukocytes using TIANamp Blood DNA Extraction Kit and following the manufacturer’s instructions (TIANGEN BIOTECH CO., LTD, Beijing, China). Genotyping of *RNF213* p.R4810K variant was performed by Taqman method (Assay ID: C_153120198_10; TaqMan SNP Genotyping Assays; Applied Biosystems) using a Roche LightCycler® 96 Real-Time PCR System (Roche, Switzerland) and analyzed with the LightCycler® 96 software. The investigators involved in genotyping were blinded from the phenotypic information. All analyses of the genotyped data were performed at the Department of Epidemiology and Health Statistics in Central South University.

### Statistical analysis

All statistical analyses were performed using SPSS 21.0 software (SPSS Inc., Chicago, IL, USA). Continuous variable (age) was presented as the mean ± standard deviation (SD). Categorical variables (hypertension, diabetes, hyperlipemia, coronary heart diseases, smoking, drinking) were presented as proportions. Categorical variables were compared using the *χ*2 test or Fisher exact test, and continuous variables were compared using Student t-tests. A *p*-value less than 0.05 was considered statistically significant.

## Results

This study totally included 715 patients with non-MMD ICASO and 507 normal individuals without known cerebrovascular diseases. Clinical characteristics are shown in Table 1. Six of 715 ICASO patients (0.84%) and 2 of the 507 normal controls (0.39%) had the *RNF213* p.R4810K variant (all heterozygotes). Even no statistically significant association was observed, the carrier rate of *RNF213* p.R4810K was higher in ICASO group than that in the normal individuals (Odds ratio, 2.14; 95% confidence interval, 0.43–10.63; *p* = 0.56).

**Table 1:** Characteristics of the participants and distribution of the RNF213 p.R4810K variant

Characteristics	ICASO	Controls
Number of the participants	715	507
Age (yrs)		
Mean ± SD	58.4 ± 12.9	37.2 ± 16.9
Range	15–89	-
Female, n (%)	253 (35.4)	377 (74.4)
Conventional risk factors, n (%)		
Hypertension	429 (60.0)	-
Diabetes	179 (25.0)	-
Hyperlipemia	290 (40.6)	-
Coronary heart diseases	79 (11.0)	-
Smoking	277 (38.7)	-
Drinking	150 (21.0)	-
*RNF213* p.R4810K genotype		
Wild type: GG (%)	709 (99.16)	505 (99.61)
Heterozygous: GA (%)	6 (0.84)	2 (0.39)
Homozygous: AA (%)	0 (0)	0 (0)
OR (95% confidence interval)	2.14 (0.43–10.63)	
*p* value	0.56	

*ICASO* intracranial major artery stenosis/occlusion, *SD* standard deviation

**Fig. 1:**
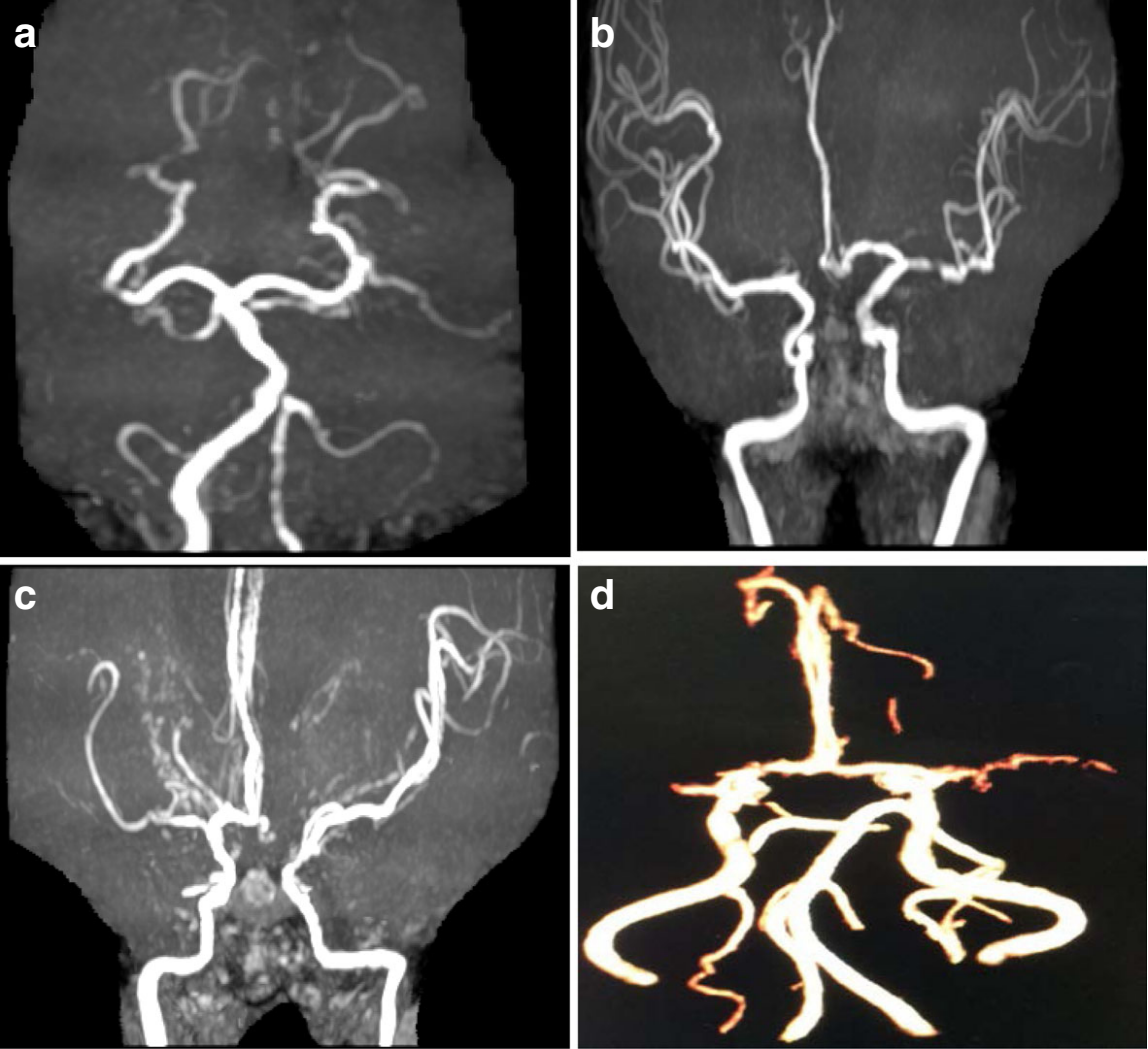
Magnetic resonance angiography (MRA) images of 4 patients with the p.R4810K variant in ICASO patients. **a** Case 1 was a 80- year- old woman with occlusion of bilateral anterior inferior cerebellar artery and stenosis of left vertebral artery. The patient was relatively elderly and had hypertention, diabetes, hyperlipidemia and coronary heart disease for many years. **b** Case 2 was a 52- year- old woman with occlusion of the A1 segment of right anterior cerebral artery and stenosis of the left middle cerebral artery. She had serious hypertension (190/100 mmHg) for 1 month. **c** Case 3 was a 42- year- old man with occlusion of the A1 segment of left anterior cerebral artery. The patient had hypertension and diabetes both for 5 years. **d** Case 4 was a 48- year- old woman with bilateral middle cerebral artery and the right anterior cerebral artery stenosis. She also had hypertension and diabetes for several years

Table 2 shows the clinical characteristics of 6 non-MMD ICASO patients with the *RNF213* p.R4810K variant. The Fig. 1 shows the MRA images of the 4 patients with ICASO identified with the p.R4810K variant (the digital MRA images of the other 2 patients with the p.R4810K variant in the ICASO group was not available due to the fact that the patients were referrals from the other hospitals). These ICASO patients showed partial stenosis or occlusion of the intracranial major artery without abnormal vascular networks in the basal ganglia on MRA. All the patients had hypertension, 4 patients had diabetes and 2 elderly patients also had coronary heart disease for more than 20 years.

**Table 2:** Clinical characteristics of 6 non-MMD ICASO patients with the RNF213 p.R4810K variant

Case	Sex	Age (yrs)	Site of stenosis/occlusion	Hypertension	Diabetes	Hyperlipemia	CHD	Smoking	Drinking
1	F	80	Occlusion of bilateral anterior inferior cerebellar artery and stenosis of left vertebral artery	5 years	2 years	2 years	20 years	-	-
2	F	52	Occlusion of the A1 segment of right anterior cerebral artery and stenosis of the left middle cerebral artery	1 month	-	-	-	-	-
3	M	42	Occlusion of the A1 segment of left anterior cerebral artery	5 years	5 years	-	-	-	-
4	F	48	Stenosis of bilateral middle cerebral artery and the right anterior cerebral artery	8 years	3 years	-	-	-	-
5	M	79	Occlusion of the left middle cerebral artery	40 years	14 years	-	23 years	-	-
6	M	63	Diffuse stenosis of the M1 segment of the left middle cerebral artery	25 years	-	-	-	10 years	-

*ICASO* intracranial major artery stenosis/occlusion, *MMD* moyamoya disease,*F* female, *M* male, *CHD* coronary heart disease

Table 3 shows the distribution of *RNF213* p.R4810K in MMD, Non-MMD ICASO and normal individuals in Japan, Korea and China. The carrier rate of *RNF213* p.R4810K in Chinese MMD and non-MMD ICASO patients was significantly lower than that in Korea and Japan.

**Table 3:** Comparision of *RNF213* p.R4810K distribution in MMD, non-MMD ICASO and normal individuals in Japan, Korea and China

Diseases	Countries	Cases						Normal individuals						OR (95%CI)^a^	Reference
		N	GG	GA	AA	Carrier rate (%)	MAF%	N	GG	GA	AA	Carrier rate (%)	MAF%		
MMD	Japan	105	17	84	4	83.81	43.81	457	438	19	0	4.16	2.08	119.3 (59.7–238.7)	[2]
		161	16	135	10	90.06	48.14	384	374	9	1	2.60	1.43	338.9 (150.3–764.2)	[1]
		204	36	153	15	82.35	44.85	283	278	5	0	1.77	0.88	259.5 (99.9–674.1)	[10]
		48	7	40	1	85.42	43.75	25	25	0	0	0.00	0.00	-	[4]
		30	10	19	1	66.67	35.00	110	108	2	0	1.82	0.91	108.0 (22.0–530.3)	[6]
		103	27	71	5	73.79	39.32	95	93	2	0	2.11	1.05	130.9 (30.2–568.1)	[8]
		78	12	64	2	84.62	43.59	-	-	-	-	-	-	-	[11]
		-	-	-	-	-	-	1474	1437	34	3	2.51	1.36	-	[5]
		-	-	-	-	-	-	4308	4248	57	3	1.39	0.73	-	[14]
		-	-	-	-	-	-	519	510	9	0	1.73	0.87	-	[15]
	Korea	38	8	30	0	78.95	39.47	223	217	6	0	2.69	1.35	135.6 (44.0–417.8)	[1]
		131	32	99	0	75.57	37.79	51	51	0	0	0.00	0.00	-	[9]
		165	40	112	13	75.76	41.82	294	286	8	0	2.72	1.36	111.7 (50.8–245.6)	[12]
		288	89	199	0	69.10	34.55	83	82	1	0	1.20	0.60	183.3 (25.1–1338.1)	[13]
		-	-	-	-	-	-	1516	1479	37	0	2.44	1.22	-	[16]
	China	52	40	11	1	23.08	12.50	100	98	2	0	2.00	1.00	14.7 (3.1–68.7)	[1]
		170	148	21	1	12.94	6.76	507	505	2	0	0.39	0.20	37.5 (8.7–161.5)	[3]
		96	87	8	1	9.38	5.21	96	95	1	0	1.04	0.52	9.8 (1.2–79.2)	[14]
		81	69	10	2	14.81	8.64	100	98	2	0	2.00	1.00	8.5 (1.8–39.3)	[16]
		255	175	78	2	31.37	16.08	300	300	0	0	0.00	0.00	-	[15]
		-	-	-	-	-	-	587	582	5	0	0.85	0.43	-	[5]
Non-MMD ICASO	Japan	41	32	8	1	21.95	12.20	25	25	0	0	0.00	0.00	-	[4]
		84	64	20	0	23.81	11.90	110	108	2	0	1.82	0.91	16.8 (3.8–74.6)	[6]
	Korea	221	144	77	0	34.84	17.42	51	51	0	0	0.00	0.00	-	[9]
		234	184	50	0	21.37	10.68	83	82	1	0	1.20	0.60	22.3 (3.0–164.1)	[13]
	China	715	709	6	0	0.84	0.42	507	505	2	0	0.39	0.20	2.14 (0.42–10.63)	This study

*MMD* moyamoya disease, *Non-MMD ICASO* non-moyamoya intracranial artey stenosis/occlusion disease, *N* sample size, *MAF* minor allele frequency

^a^Odds ratio (95% confidence interval) under dominant model.- can’t calculate

## Discussion

In this study, only few of Chinese ICASO patients (6/715, 0.84%) carried *RNF213* p.R4810K variant, which was significantly lower than that in Korea or Japan. Genetic heterogeneity of ICASO in different population was highly indicated.

*RNF213* was a susceptibility gene for MMD [25]. Previous studies showed that in Japan and Korea, the founder variant *RNF213* p.R4810K was much more frequent in MMD patients (~80%) than in the general population (~1.0%), significantly increased MMD risk (ORs > 100) [1, 2, 5, 8, 9, 25]. However, as a susceptibility gene, Chinese MMD patients have significantly different genetic architecture. The carrier rate of *RNF213* p.R4810K in Chinese MMD and general population is about 20 ~ 30% and 0.3% respectively, accounting for less part of MMD risk [1, 3, 5, 17–19]. The genetic result is consistent with the unique epidemiological and clinical characteristics of Chinese MMD. In China, no significant difference in sex distribution of MMD, a female predominance is not observed compared to Japan and South Korea. Moreover, the familial occurrence of MMD is lower and the symptoms at the onset are different from those in Japan and South Korea [28]. Genetic heterogeneity is proposed to be partially responsible for the different clinical features of MMD in different ethnicities. It has been proposed that other rare variants of *RNF213* may be causative mutations for MMD patients. Zhang et al. systematically investigated *RNF213* variants of 255 Chinese MMD, revealing that p.R4810K was identified in 31.4% MMD patients and additional 25 rare variants (absent in controls) were identified in 10.6% of patients without p.R4810K variant. Segregation analysis supported the association between MMD and 2 novel variants (p.H4014T and p.R4160Q) [18]. Similar with MMD, genetic heterogeneity of ICASO in different population was indicated. In previous Japanese and Korean studies [4, 6, 13], a particular subset of ICASO mainly diagnosed as atherosclerosis, associated with *RNF213* p.R4810K variant (odds ratios, 16.8 and 22.3; 95% confidence intervals, 3.81–74.5 and 3.0–164.1; both *p* < 0.0001 for Japanese and Korean, respectively). These findings strongly indicate that some cases of ICASO ascribed to unknown etiology or atherosclerosis might be caused by *RNF213* p.R4810K variant. They proposed that *RNF213* p.R4810K variant could contribute to the high prevalence of intracranial atherosclerotic stroke in Asians. However, in our study, only 0.84% Chinese ICASO patients (6/715) carried *RNF213* p.R4810K variant, no statistically significant association of *RNF213* p.R4810K variant with ICASO was observed. *RNF213* p.R4810K variant is unlikely playing major role in Chinese ICASO. Population-specific variants might contribute a lot to the pathogenesis. In addition, it has been widely accepted that MMD and ICASO is caused by both genetic and environmental factors. In this study, all 6 ICASO patients with *RNF213* p.R4810K variant had hypertension and 4 patients had diabetes. Other unknown genetic and environmental factors may trigger ICASO together. Further systematically investigate Chinese-specific genetic variants of ICASO is needed.

The limitations of this study should be mentioned. First, the analysis of the *RNF213* variant was exclusively focused on the *RNF213* p.R4810K variant. The other *RNF213* variants such as p.H4014T and p.R4160Q were not evaluated. Further comprehensive genetic analysis of *RNF213* and other potential genes is necessary to determine whether patients with ICASO without the p.R4810K variant have other variants or not. Second, because the strictly matched cerebrovascular disease-free controls were not available in this study, we used the allele frequency of p.R4810K in previous published control population as control in the association analysis. This may perturb the association results. However, such an approach could be justifiable for rare variants and could not perturb the results significantly, especially in the study with a low carrier rate of target variant in the case group. Third, due to the allele frequency of *RNF213* R4810K was low in both case and normal individual group in China, statistic power might be low when exploring the association between *RNF213* p.R4810K variant and ICASO in this study. Relevant studies with larger sample sizes are needed to validate our findings.

## Conclusion

Even no statistical significance, the carrier rate of *RNF213* p.R4810K was higher in ICASO group than that in the normal individuals in China. However, the carrier rate of *RNF213* p.R4810K in Chinese ICASO patients was significantly lower than that in Korea or Japan. Population genetic heterogeneity was highly indicated. Further systematic genetic epidemiology studies with larger sample sizes focusing on Chinese-specific genetic variants and environmental risk factors of ICASO are needed.

## Abbreviations

ICASO: Non-moyamoya intracranial major artery stenosis/occlusion
MMD: Moyamoya disease
*RNF213*: The ring finger protein 213
